# Production of Pyracantha Polysaccharide-Iron(III) Complex and Its Biologic Activity

**DOI:** 10.3390/molecules26071949

**Published:** 2021-03-30

**Authors:** Wan-Fen Li, Hao-Hai Ma, Shuai Yuan, Xi-Feng Zhang

**Affiliations:** College of Veterinary Medicine, Qingdao Agricultural University, Qingdao 266109, China; liwfqd1981@163.com (W.-F.L.); mahaohai741@163.com (H.-H.M.); arbeit19960413@163.com (S.Y.)

**Keywords:** polysaccharides, polysaccharide iron, response surface optimisation, antioxidant

## Abstract

In this study, the optimum synthetic process of the Pyracantha polysaccharide-iron (PPI) complex was studied via response surface methodology (RSM). Its antioxidant and anti-cancer activities were also investigated. It was demonstrated that the optimal conditions for the synthetic process of the complex were as follows: a pH of 8.9, a reaction temperature of 70 °C and a trisodium citrate:polysaccharide ratio of 1:2. PPI were analysis by UV, FTIR, SEM, CD, XRD, TGA and NMR. PPI was able to scavenge the metal ion, ABTS and free radicals of the superoxide anion, demonstrating its potential antioxidant activity. PPI was found to display cytotoxicity to Skov3 cells, as shown by its ability to induce apoptosis and alter gene expression in Skov3 cells. These findings show than PPI may represent a novel antioxidant and chemotherapeutic drug.

## 1. Introduction

Polysaccharides are ubiquitous biological macromolecules that are involved in myriad physiological human processes. These molecules have been documented to enhance the body’s immunity, prevent tumours, and possess antiviral properties. Modern day formulations of polysaccharides in combination with metals and non-metals have been shown to possess good functional qualities [1]. Iron is an essential trace element in the human body and participates in several enzymatic reactions in the body, with its most well-known role being oxygen transportation as well as the maintenance of cellular metabolism [2]. A polysaccharide iron complex can be used as a supplement that has very stable properties. Valent iron complexes that enter the body produce free radicals, which contribute to cell membrane damage [3]. Oral consumption of iron complexes also leads to a host of side effects, such as nausea and vomiting [4]. Polysaccharide iron complexes are usually complexes of ferric ions [5,6]. These compounds have a relatively high iron concentration and can be soluble and non-toxic at physiological pH values, making them an efficacious iron supplement in the body [7].

Many studies have shown that the polysaccharide iron complex possesses high stability, water solubility and a good absorption rate [8,9,10]. It also has a safer adverse event profile compared to ferrous sulphate. Given these excellent properties, the polysaccharide iron complex has gained recognition as a potential treatment for anaemia. Chemical synthesis and simulated biomineralisation are the main methods for the preparation of polysaccharide iron complexes, with the former method being more common. The polysaccharide iron complex is a chelation consisting of polysaccharides and iron that does not cause gastrointestinal discomfort, as the ferric iron is bound and not in its free state. In the body, ferric iron is absorbed and reduced to ferrous ion. Polysaccharide chelated iron exists mostly in a ferric state. The polysaccharide is primarily extracted from plants and has immunomodulatory effects. The *Pyracantha fortuneana* fruit is a high-quality medicinal and edible natural plant resource that contains several active ingredients. It is rich in carbohydrates and the total soluble sugar content of the flesh of the fruit has been reported to be between 10.59–13.40% [11]. Polysaccharides derived from the *Pyracantha fortuneana* fruit are promising compounds to be used in the formulation of polysaccharide iron complexes. We have completed the extraction and structural characterization of polysaccharides of *Pyracantha fortuneana* (PSPF) [12].

In this study, response surface methodology (RSM) was used to study the preparation of PPI, and the antioxidant effect of the *Pyracantha* polysaccharide-iron(III) complex was also studied. Human ovarian carcinoma cells (Skov3) was used as a model cell to investigate the anti-cancer activity of this compound.

## 2. Results and Discussion

### 2.1. Establishment of a Standard Curve for Iron

Based on the absorbance of different concentration gradients at 510 nm, the regression equation is given as the following equation: y = 0.0982x + 0.0041 (R^2^ = 0.999). There was a good linear relationship from 0.4–2.8 μg/mL.

### 2.2. Response Surface Optimization of PPI Complexsynthesis Conditions

**Figure 1:**
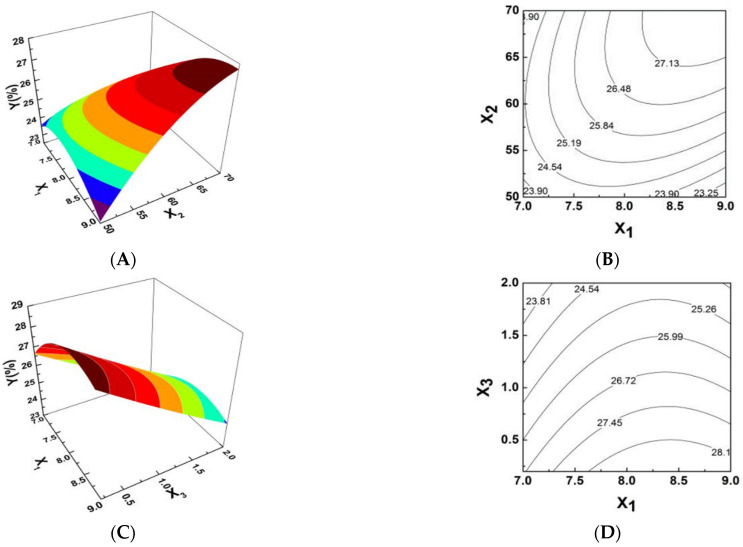

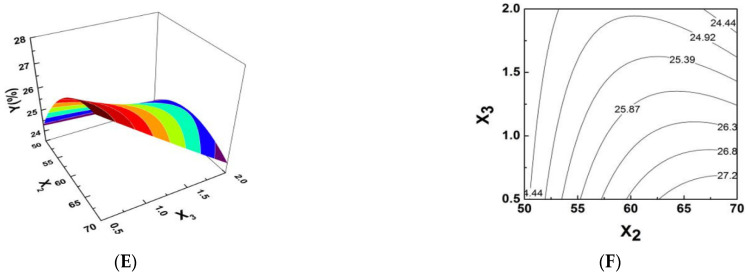
Response surface plots showing the effects of variables on the yield of PPI. (**A**,**B**) Plots of the effect of pH, temperature and their reciprocal interactions on the iron content (%). (**C**,**D**) Plots of the effect of pH, ratio of trisodium citrate to polysaccharide and their reciprocal interactions on the iron content (%). (**E**,**F**) Plots of the effect of temperature, ratio of trisodium citrate to polysaccharide and their reciprocal interactions on the iron content (%).

Response surfaces were plotted using Design Expert software [13]. As shown in Figure 1, response surface plots and the respective contour plots demonstrated the effects of two factors and were used to obtain the optimum conditions for the production of PPI. The interactions amongst the factors in the response surface can be intuitively shown by the degree of contour density in the contour map and the steepness of the response surface map in Figure 1. Figure 1A,B represents the effects of different pH (X_1_) and different extraction temperatures (X_2_) on complex synthesis at a given amount of catalyst. It shows that the 3-D plot and the contour plot described the effect of pH surface as relatively steep with a more intensive contour.

Comparatively, the contour plot for effect of reaction temperature of the surface was smooth with relatively sparse contours, indicating that pH levels had a more significant effect on PPI synthesis. Figure 1C,D shows the 3-D plot and the contour plot at ratio of catalyst to *Pyracantha fortuneana polysaccharides* (PSPF) and pH. The effect of catalyst surface is relatively steep, dense and possesses obvious contours while the contour plot of the pH effect is relatively smooth with sparse contours, indicating that the catalysts used has a more significant effect on PPI synthesis. The 3-D plot and the contour plot based on independent variable ratio of catalyst to polysaccharides and reaction temperature are shown in Figure 1E,F. The contour plot of the effect of the catalyst surface is relatively steep, with more intensive contours, whereas that of the effect of reaction temperature has relatively smooth sparse contours, also indicating the more significant effect of catalysts on PPI synthesis. As shown in Figure 1, the highest point in the graph is also the centre point of the smallest ellipse in the contour line [14]. As shown in the figure above, the contour diagram of polysaccharide iron shows strong interactions amongst the various factors. This conclusion is consistent with the ANOVA results in Table 1.

Through the response surface software, the optimal reaction conditions for the production of PPI were 70 °C pH 8.9 and ratio of catalyst to polysaccharide 0.5. Under the optimal conditions, we were able to achieve an iron content of PPI of 30.76% (*n* = 5), close to the predicted iron content 30.81%. The model was proven to be valid and can be used as an established protocol for PPI synthesis.

The ANOVA results are demonstrated in Table 1. The significance analysis of experiment results were obtained using the F-value and *p*-value [14]. A large F-value and a small *p*-value indicate a more significant effect on the response variable [15]. According to Table 2, the F-value was 89.09, and the *p*-value of lack of fit was <0. 0001, indicating that the lack of fit was not significant. Values of ‘Prob. > F’, more than 0.0500 indicate not significant model terms. In Table 2, the variables with a significant effect on the yield of PPI were the linear coefficients (X_1_, X_2_, X_3_), the quadratic term coefficient (X_1_^2^, X_2_^2^), and the cross product coefficients (X_1×2_, X_2_X_3_).

The model fit was checked against the determination coefficient (R^2^) [16]. In this study, the value of R^2^_Adj_ (98.02%) and the R^2^_pred_ of 92.18% were in reasonable agreement with the R^2^_Adj_ of 98.02%. This indicates that the fitted quadratic model accounts for more than 98.02% of the variations in the experimental data. The coefficient of determination R^2^ (99.18%) shows that the model has a good fitting degree and can be used to analyse and predict the absorbance value of PPI. ‘Adeq. Precision’ was used to measure the signal to noise ratik. An ‘Adeq. Precision’ of 33.858 is high enough to indicate that the model is predictive regarding experimental results.

### 2.3. Characterisation of PPI

**Figure 2:**
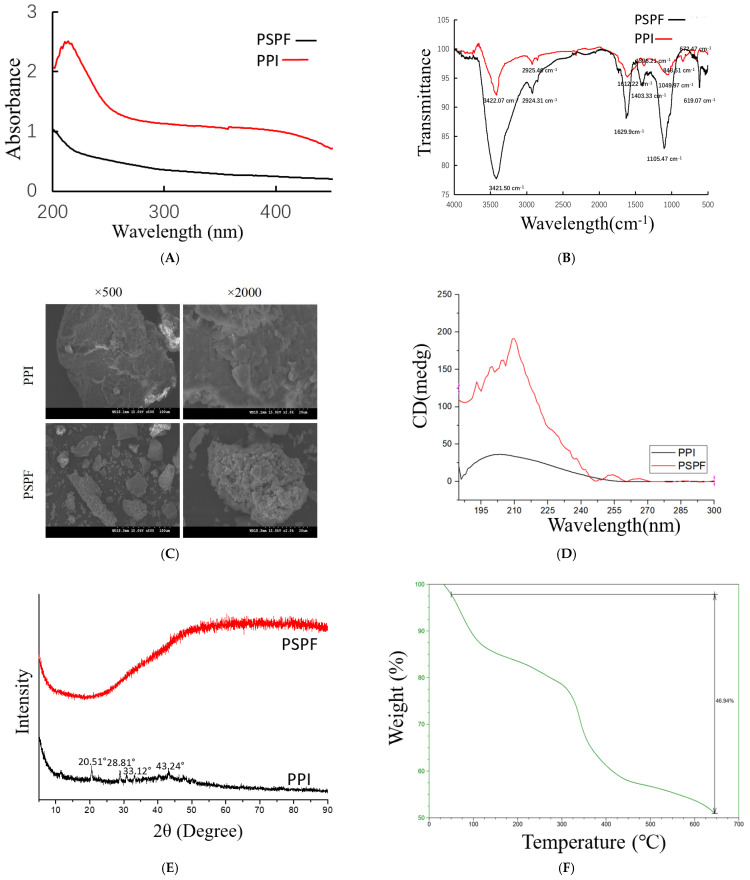

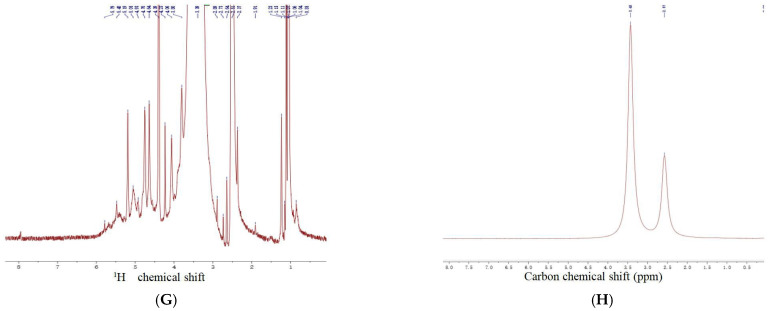
Analysis of PSPF and PPI (**A)**. UV analysis of PSFP and PPI. (**B**) FT-IR spectrum of PSFP and PPI. (**C**) SEM images of the PPI and PSPF. (**D**).CD spectra of the PPI and PSPF. (**E**) X-ray diffraction (XRD) pattern of PSPF and PPI. (**F**) TG curves of PSPF under nitrogen condition. ^1^H NMR spectra, (**G**) PSPF, (**H**) PPI.

Figure 2A depicts reactions between PSPF and PPI. The absorption of PPI was in accordance with the previous literature and ranged from 450 nm to 200 nm, suggesting that the iron core in PPI was Fe-OOH [17]. At the same concentration, compared with indicates that PSPF reacts with iron to form a PSPF, the absorption intensity of PPI in ultraviolet region is obviously increased, which complex without free substance [18]. Figure 2B demonstrates the infrared spectra of PSPF and PPI. The infrared spectra of the two samples are relatively similar, with both displaying strong absorption peaks near 3500–4000 cm^−1^, which is due to the strong hydrogen bonding of multiple -OH in polysaccharide molecules. They belong to the absorption peaks of light stretching vibrations in the PSPF. As shown in Figure 2B, PPI also possessed another peak at 672.47 cm^−1^. Taken together, this suggests that the core of the polysaccharide iron complex was a β-FeOOH nucleus, which is consistent with the report of Marshall [19].

The SEM images of PPI and PSPF at different magnifications (2000× and 500×) are illustrated in Figure 2C. The surfaces of PPI and PSPF had obvious variations in size and shape. The image at a low magnification shows that the surface morphology of PSPF was characterised by a small size and homogeneous dispersal. The surface of PPI presents smooth and irregular flaky structure, and the slightly uneven surface of PSPF is more obvious than PPI.

CD was an effective method to investigate the three-dimensional structure of compounds. A new positive Cotton effect appeared at 215 nm with increasing intensity (Figure 2D), which might suggest that the structural asymmetry in PPI was enhanced, which might be attributed to the charge transfer interaction between iron ions and the carboxyl group in polysaccharide chains [20].

XRD is an effective method for analysing the microstructure of crystalline materials and some non-crystalline materials. X-ray diffraction patterns of PPI and PSPF are shown in Figure 2E. The XRD pattern of PPI presented strong diffraction peaks at 20–45° compared to PSPF. The diffraction peaks of PPI were at 20.51°, 28.81°, 33.12°, and 43.24°. Additionally, the XRD results confirmed that a new compound, PPI, was formed and with a large amount of iron(III) [21].

The thermal gravimetric curve of the PPI is presented in Figure 2F, which shows the degradation pattern of PPI. As shown in Figure 2F, there were three weight loss events for the PPI. The first weight loss of PPI occurred in the temperature range of 25–200 °C and the loss rate was 18%. The next weight loss of PPI occurred at 200–380 °C and the loss rate was up to 20%. The third weight loss of PPI occurred at 380–650 °C and the loss rate was 15%. Therefore, it is relatively stable.

^1^H NMR spectroscopy were performed for analysis of the structure of PSPF and PPI. The ^1^HNMR spectrum of PSPF (Figure 2G) revealed different linkage patterns at 5.0–4.0 ppm. However, the complete signal pattern of PSPF was missing in the spectrum of PPI (Figure 2H), This might be due to the relatively large, blinded region around high-spin (S = 5/2) iron(III) centres, which was the same of the reports of the Bertini group [22]. In the metal centre, ligand signals were too broad to be detectable due to shortened relaxation times [23].

### 2.4. Antioxidant Activity of PPI

**Figure 3:**
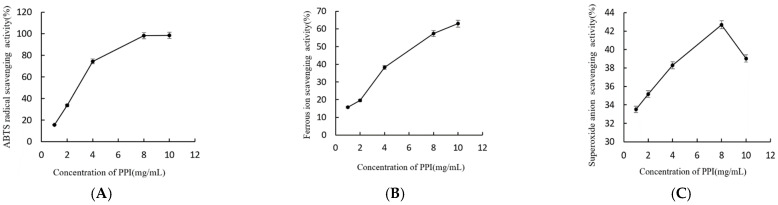
Antioxidant activity of PPI (**A**). Scavenging effects of PPI on ABTS radical. (**B**). Scavenging effects of PPI on ferrous ion scavenging. (**C**). Scavenging effects of PPI on superoxide anions.

As shown in Figure 3, PPI exhibited apparent antioxidant activity. The antioxidant activity of PPI through TEAC (on ABTS^+^ radical scavenging activity) is depicted in Figure 3A. The scavenging activity increased with increasing concentrations of PPI. The maximum clearance rate was up to 98.5%, when the concentration of PPI reached 10 mg/mL. Metal chelating activity has long been regarded as a strong antioxidant mechanism given its ability to reduce lipid peroxidation in metal catalysts. The antioxidant activity of PPI through metal ion scavenging activity is shown in Figure 3B. PPI exhibited apparent binding capacity in a concentration-dependent manner. The maximum clearance rate was 63%, when the concentration of PPI reached 10 mg/mL. The scavenging activities of PPI on superoxide anion are presented in Figure 3C. The superoxide radical scavenging activities of PPI were concentration-dependent when the mass concentration of PPI was 1–8 mg/mL and the maximum clearance rate was 42.69%. On the other hand, super-oxide-radical scavenging activities of PPI were decreased when the mass concentration of PPI was 10 mg/mL. After combining with iron ions, PPI enhanced the ABTS scavenging activity much more than PSPF, but there was no significant change in ferrous ion and superoxide anion scavenging activity.

### 2.5. Effect of PPI on Cell Viability and Cell Morphology

**Figure 4:**
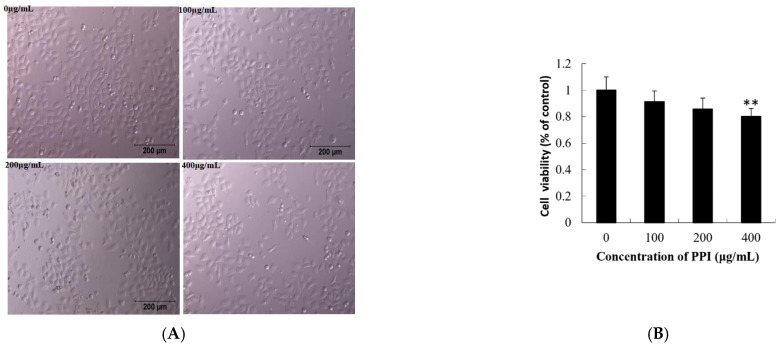

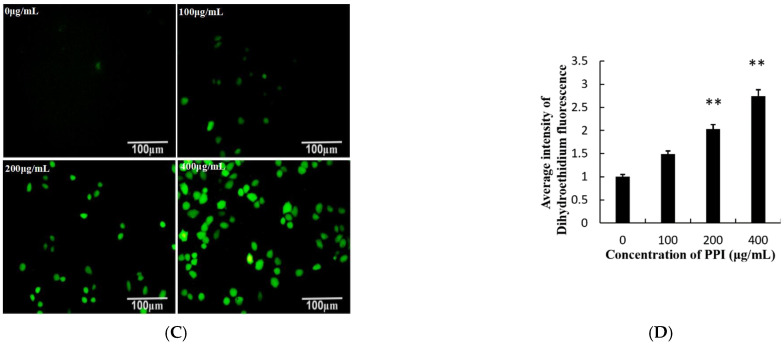
Effects of PPI on cell viability and evaluation of ROS level. (**A**). Phase contrast microscopy showing the morphology of Skov3 cells after PPI treatment for 24 h. Scale bars = 200 µm. (**B**). Data for Skov3 cells. (**C**). Intracellular ROS levels were measured with fluorescence imaging using the DCFH-DA probe in cells cultured in the presence of PPI (0, 100, 200 and 400 µg/mL) for 24 h. Scale bars = 100 µm. (**D**). The average intensity of fluorescence in Skov3 cells. Data are expressed as mean ± SD of three independent experiments performed in triplicate. ** *p* < 0.01.

As shown in Figure 4A,B, the viability of Skov3 cells was significantly decreased after 24 h of exposure to PPI. Cell viability was affected in a dose-dependent manner, with the viability of Skov3 cells decreasing significantly as concentrations increased from 100 to 400 µg/mL PPI (*p* < 0.05; Figure 4B). Compared with PSPF, PPI has weaker inhibitory activity on cancer cell proliferation. The viability of human ovarian cancer cells decreased significantly exposure to 100 and 200 μg/mL PSPF [12].

### 2.6. PPI Increased Levels of Reactive Oxygen Species

Studies have shown that ROS are closely related to apoptosis, as increasing the ROS level leads to a decrease in the level of cellular antioxidants, resulting in overall oxidative damage to cellular components [24,25]. ROS was detected using a fluorescent DCFH-DA probe. As shown in Figure 4C,D, the results indicate that the intercellular ROS induced by PPI in Skov3 cells was upregulated in a dose-dependent manner. Simultaneously, the results show that PPI was responsible for cell death and enhanced ROS production.

### 2.7. Loss of Mitochondrial Membrane Potential (∆ψm) and Apoptosis Induction

**Figure 5:**
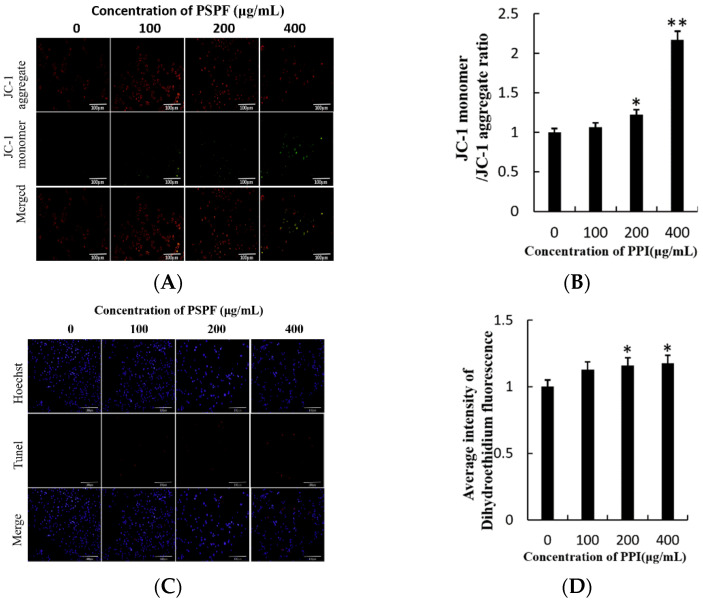
Loss of mitochondrial membrane potential (∆ψm) and TUNEL assay. (**A**). The ∆ψm was evaluated using JC-1 in treated cells. Red fluorescence indicates JC-1 aggregates within mitochondria in healthy cells, whereas green fluorescence indicates JC-1 monomers in the cytoplasm and loss of ∆ψm. Scale bars = 100 µm. (**B**). Ratio of JC-1 monomers to JC-1 aggregates. (**C**). TUNEL assay in treated cells, scale bars = 100 µm. (**D**). Average intensity of TUNEL fluorescence in Skov3 cells. * *p* < 0.05, ** *p* < 0.01.

**Figure 6:**
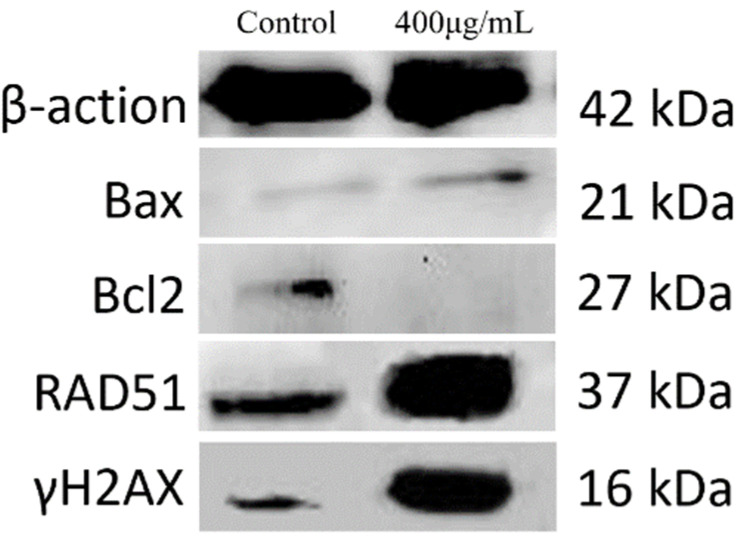
Western blotting. Bax, Bcl2, RAD51, γH2AX and p53 expression in Skov3 cells. Cells were treated with PPI at 400 µg/mL for 24 h.

The decline in ∆Ψm is an early marker of apoptosis. Cells have been shown to enter an irreversible apoptosis process if ∆Ψm is lost. JC-1, the ideal fluorescent probe for ∆Ψm detection, can easily be detected by the transition from red to green fluorescence [26,27]. The intensity of JC-1 was significantly decreased (*p* < 0.01) in the groups treated with PPI in a dose-dependent manner (Figure 5A,B). DNA degradation in the early stage of apoptosis was evaluated using the TUNEL assay [28]. Compared with the control group, TUNEL-positive cells and fluorescence intensity were high in the groups treated with higher concentrations of PPI (Figure 5C,D), indicating more pronounced apoptosis. Furthermore, the expression of proteins related to apoptosis were detected by Western blot analysis (see Section 2.9). The expression of Bax protein was significantly up-regulated while Bcl-2 was significantly down-regulated in PPI-treated Skov3 cells. Additionally, the expression of γ-H2AX and RAD51, which were detected by immunofluorescence, were also significantly up-regulated (Figure 6). Compared with PSPF, PPI has weaker ability to induce cell apoptosis [12].

### 2.8. PPI Induces Nuclear DNA Breakage

**Figure 7:**
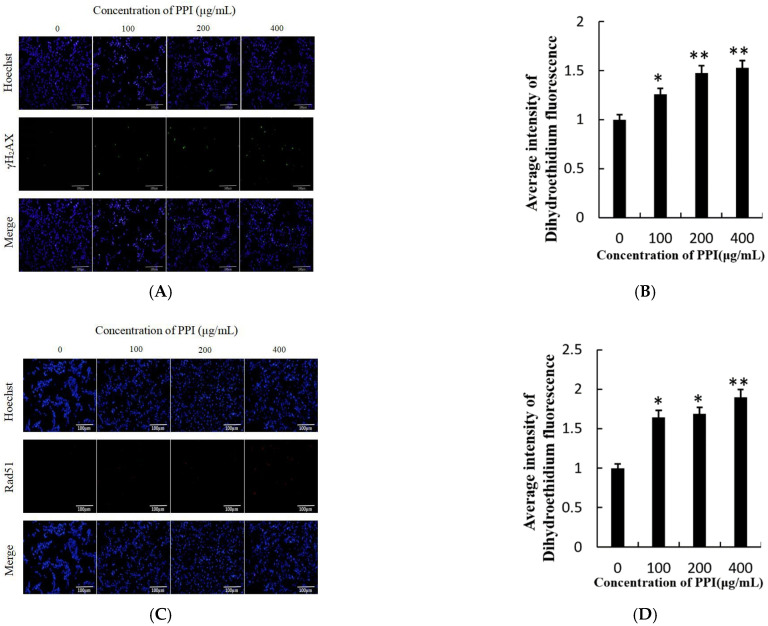
Nuclear DNA damage in Skov3 cells after CPPI treatment using immunocytofluorescence with γ-H2AX and Rad51 antibody. (**A**). Immunocytofluorescence with γ-H2AX in treated cells. (**B**). Average intensity of green fluorescence in Skov3 cells. (**C**) Immunocytofluorescence with Rad51 in treated cells. (**D**). Average intensity of red fluorescence in Skov3 cells. Scale bars = 100 µm. * *p* < 0.05, ** *p* < 0.01.

Apoptosis is always accompanied by DNA damage. γ-H2AX and Rad51, both markers of DNA damage, play major roles in the repair process after DNA damage [29,30]. Levels of phosphorylated γ-H2AX in cells exposed with PPI for 24 h were measured using immunofluorescent images (Figure 7A). Green fluorescence, which represents γ-H2AX, was remarkably enhanced in PPI-treated samples in a dose-dependent manner (Figure 7B, *p* < 0.01). The presence of DNA damage was further confirmed by RAD51 staining (Figure 7C,D). We further analysed their expression using WB. The expression levels of γ-H2AX and RAD51 all were up-regulated in the 400 μg/mL PPI treatment group compared to the control group (Figure 6).

### 2.9. PPI Affected Gene Expression in Skov3 Cells

**Figure 8:**
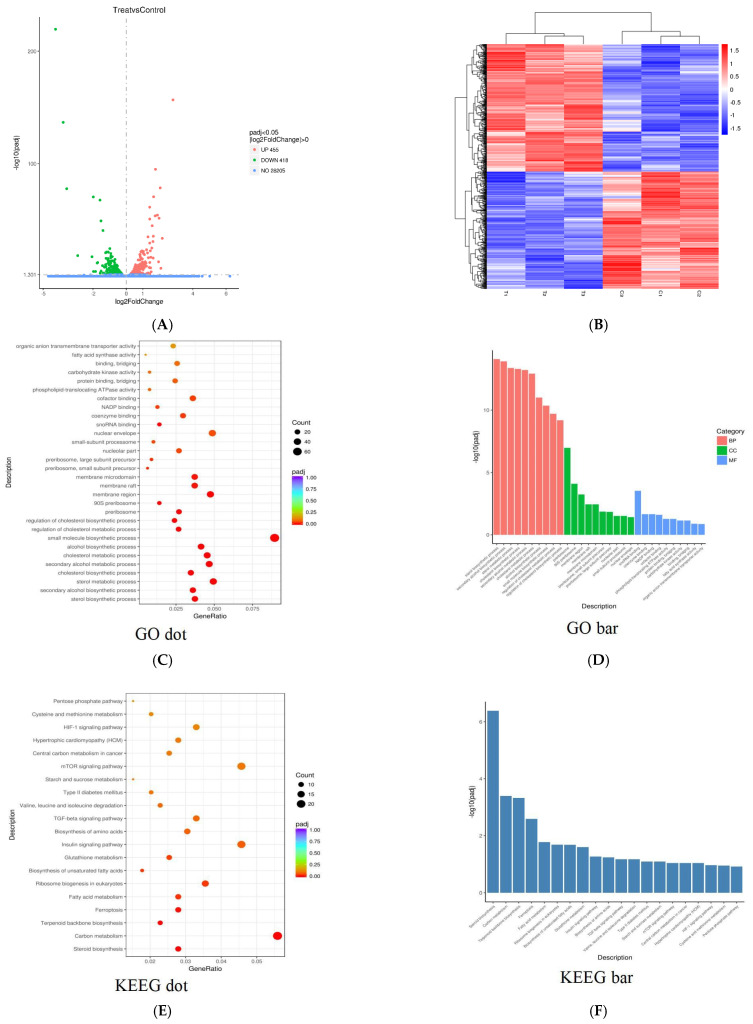
Gene expression in PPI-treated cells. Scatter plot of PPJ-treated Skov3 cell gene expression profiling. (**A**) The 455 up-regulated genes and 418 down-regulated genes in the treated group are plotted in green and red, respectively. (**B**) Hierarchical clustering analyses of the differentially expressed genes in two replicates of the two groups. (**C**) Dot plots of overrepresented gene ontology (GO) terms in molecular function (MF), biological process (BP) and cellular component (CC) categories. (**D**) Bar plots of overrepresented GO terms in MF, BP and CC categories. (**E**) Dot plots of Kyoto Encyclopaedia of Genes and Genomes (KEGG) terms. (**F**) Bar plots of KEGG terms. Top ten significant terms are shown in each category (*p* < 0.05).

In order to reveal the influence of PPI exposure on gene expression in Skov3 cell, Skov3 cell mRNA was examined by high-throughput sequencing. As shown in Figure 8A, a total of 29,078 genes were detected. There were 873 genes which were significantly different between control and PPI-treated groups. A total of 455 genes were significantly up-regulated and 418 were down-regulated after PPI treatment. The scatter plot is presented as a log2 of fold change of signal intensity and blue spots represent genes expressed at similar levels; the up-regulated genes in the PPI-treated group are marked in red, and the down-regulated genes are marked in green. The hierarchical clustering analyses of the 863 differentially-expressed genes (DEGs) is shown in Figure 8B. Sterol biosynthetic process, secondary alcohol biosynthesis process and sterol metabolic process were the mainly enriched DEGs in BP; the DEGs in CC were related to the preribosome, 90s preribosome and membrane region, and snoRNA binding and coenzyme binding were significant DEGs in MF between the control and PPI-treated group (Figure 8C,D). Through KEGG analysis, steroid biosynthesis, carbon metabolism and terpenoid backbone biosynthesis were the most significantly altered signalling pathways between the control and treated groups (Figure 8E,F). PPI also causes cell ferroptosis, a new cell death mode discovered in recent years (Figure 8E,F).

## 3. Conclusions

In this study, PPI was synthesised and studied in detail. The synthesis of PPI was successfully optimised using RSM. The optimal conditions that achieved maximum complex yield were a reaction temperature of 70 °C, pH 8.9 and a catalyst to polysaccharide ratio of 0.5. Under the optimal conditions, the PPI produced was a reddish-brown powder with an iron content of PPIC that reached 30.76%, which suggests that the model was satisfactory and accurate. In addition, the antioxidant study found that PPIs have a clear role in scavenging superoxide radicals, metal ions and ABTS radicals. The results indicate that PPI had antioxidant activities in the in vitro assays. Additionally, we also verified that PPI could induce significant cytotoxicity, as evidenced by an increase in intracellular ROS, mitochondrial membrane potential disruption and consequent DNA damage. Moreover, PPI exposure significantly altered the cancer-related gene expression pattern of Skov3 cells in vitro, which suggests that PPI could be a potential drug for anticancer therapy.

## 4. Materials and Methods

### 4.1. Drugs and Reagents

*Pyracantha fortuneana* polysaccharide (PSPF) were extracted and purified in our laboratory [12], sodium citrate tribasic, 2,20-azino-bis (3-ethylbenzthiazoline-6-sulfonic) acid (ABTS) was provided by Sigma (St. Louis, MO, USA). All other chemicals and reagents were purchased locally and were of analytical grade.

### 4.2. Preparation of Polysaccharide

*Pyracantha fortuneana* polysaccharide (PSPF) was extracted with water and alcohol precipitation under the following conditions: extraction time of 2.1 h, 61.5 °C, ratio of water to raw material = 36.3 [12]. D101 macroporous adsorption resin was used for pigment removal with protein removed by the Sevage method. The resultant polysaccharide was collected, concentrated, dialysed and lyophilised for purification.

### 4.3. Single Factor Experimental Data Analysis

Based on a literature review of the synthesis process of polysaccharide iron, the main factors affecting polysaccharide iron are as follows: citric acid, the ratio of catalyst to polysaccharide, temperature, pH and reaction time [31]. In this study, pH, temperature and the ratio of catalyst to polysaccharide were selected as three independent variables. As shown in Table 3, pH = 8, temperature = 60 °C and the ratio of catalyst to polysaccharide = 1.25 were each at their optimal values.

### 4.4. Synthesis of Pyracantha Polysaccharide-Iron(III) Complex (PPI)

PPI was synthesised chemically. Other methods of PPI synthesis that have previously been documented include the ammonium sulphate method and the composite membrane method; Tang and Liu have prepared the polysaccharide iron complex using a chemical synthesis method [32].

### 4.5. Response Surface Design

In this experiment, the iron content of PPI was used as an evaluation index. The PPI at points based on the experimental design are shown in Table 2. The whole design consisted of 17 experimental points carried out at random. The best fitting model was the result of RSM as determined via regression using Design Expert software (v.8.0.6.1 trial, Stat-Ease Inc., Minneapolis, MN, USA). At the bottom of Table 1, the fitted quadratic polynomial equation is given as follows: Y = 0.52 + 0.0.015X_1_ + 0.026X_2_ − −0.031X_3_ + 0.025X_1_X_2_ − 2.35*10^−3^X_1_X_3_ − 0.026X_2_X_3_ − 0.022X_1_^2^ − 0.017X_2_^2^ + 9.1*10^−4^X_3_^2^.

### 4.6. Determination of PPI

#### 4.6.1. Establishment of Standard Curve for Iron

The two valent iron standard solution configuration is as follows: a standard iron solution was prepared by dissolving 0.7025 g of (NH_4_)Fe(SO_4_)_2_·H_2_O in 1000 mL of distilled water with the addition of 2 mL of hydrochloric acid iron. Then, 0, 1, 2, 3, 4, 5, 6 and 7 mL of iron standard solution was added to 1.75 mL of ascorbic acid and 2.5 mL of phenanthroline. After 10 min, the UV absorbance of sample was assessed at 510 nm. Finally, the corresponding standard curve was obtained. The calibration curve was y = 0.0982x + 0.0041, R^2^ = 0.9999.

#### 4.6.2. Determination of the Iron Content in PPI

The iron content was measured using the phenanthroline colorimetric method [33]. For this, 0.05 g of PPI was dissolved in 50 mL of water, then 1.0 mL of the sample was mixed with 2.5 mL of 10% ascorbic acid solution and 5 mL of 0.1% o-phenanthroline solution. Finally, the solution was adjusted to 50 mL with water. After 3 h, the UV absorbance of the sample was assessed at 510 nm. The iron content of PPI was calculated using the calibration curve.

#### 4.6.3. Experimental Design

RSM is a mathematical and statistical technique to optimise the available parameters through the least number of experiments and to analyse the interaction between these parameters [34,35]. In this study, RSM was adopted to optimise the process conditions for PPI production. The three-level Box–Behnken design (BBD) was used to evaluate three independent variables on the basis of single factor experiments: pH (X_1_), temperature (X_2_) and the ratio of catalyst to PSPF (X_3_). The iron content of PPI was selected as the response. The variables and their respective levels are presented in Table 1.

ANOVA was used for the statistical analysis. *p*-values less than 0.05 were considered statistically significant. ANOVA, regression analysis and the response surface rendering method were used to fit the quadratic polynomial equation of all response variables in order to obtain the optimum conditions for complex formation.

#### 4.6.4. Antioxidant Activities of PPI

The antioxidant activities of PPI were assessed based on previously established methods [36,37,38,39]. For this, 1, 2, 4, 8 and 10 mg/mL PPI was prepared in water. The scavenging ability for pyrogallol autoxidation was calculated: inhibition rate (%) = (1 − (A_1_ − A_2_)/A_0_) × 100%, where A_0_ is the absorbance of the control (water instead of sample), A_1_ is the absorbance of the sample and A_2_ is the absorbance of the sample with anhydrous ethanol instead of pyrogallol.

### 4.7. Characterisation of PPI

#### 4.7.1. UV and FTIR Analysis

PSPF and PPI powder were dissolved in 50 mL of water. The absorbance was measured from 200 to 450 nm using an ultraviolet spectrum scan. Absorptions of PSPF and PPI were identified by the FTIR spectrum. Approximately 2 mg of PSPF and PPI powder were weighed and mixed with KBr powder, ground and pressed for FTIR measurements through spectrometry (PerkinElmer, Spectrum 400, Waltham, MA, USA).

#### 4.7.2. SEM Analysis

The surface morphology of PPI and PSPF were analysed by scanning electron microscopy (Hitachi, Tokyo, Japan). With the help of double-sided tape, the sample was placed on a brass stub and observed after coating with gold in a vacuum by a sputter coater.

#### 4.7.3. Circular Dichroism (CD) Analysis

The CD spectra of PSPF and PPI solutions (1.0 mg/mL) were measured on a Chirascan V100 CD (Applied Photophysics, Leatherhead, UK) spectropolarimeter at 25 °C. Each CD spectrum was the accumulation of three scans at 100 nm/min with a 1 nm slit width and a time constant of 1 s. Data were collected from 185 to 300 nm at 1 nm intervals.

#### 4.7.4. Structural Characteristics of PSPF and PPI

X-ray diffraction (XRD) analysis was performed with an X-ray diffractometer (D8ADVANCE, Bruker, Karlsruhe, Germany). The X-ray diffractometer was operated at 40 kV and 30 mA produced with a Cu-κα radiation. The XRD patterns were recorded over 5–90° (θ being the angle of diffraction) at a rate of 4°/min.

#### 4.7.5. Thermogravimetric Analysis (TGA)

A simultaneous thermal analyser (STA449F3, Netzsch Corporation, Selb, Germany) was applied to determine the thermal stability of PPI. Approximately 10 mg of PPI powder was placed in a platinum crucible and heated from 30 to 650 °C at a rate of 10 °C/min in a nitrogen atmosphere with a flow rate of 50 mL/min.

#### 4.7.6. Nuclear Magnetic Resonance (NMR) Analysis

For NMR measurements, PPI and PSPF powder were completely dissolved in 0.5 mL of dimethyl-d6 sulfoxide (D-DMSO) and transferred into an NMR tube. The ^1^H spectra experiments were recorded at room temperature at 500 MHz on a spectrometer (AVIII500, Bruker, Fällanden, Switzerland).

#### 4.7.7. Cytotoxicity of PPI Assay and ROS Assay

Skov3 (human ovarian carcinoma cells) were cultured in DMEM/F12 medium + FBS (10%) and antibiotics (100 U/mL penicillin together with 100 µg/mL streptomycin) in a 5% CO_2_ atmosphere at 37 °C in humidified incubator. Different concentrations of PPI (0, 100, 200, 400 μg/mL) were used to treat cells for 24 h, and the MTT assay was used to evaluate the cytotoxicity of PPI [40]. The S0033 detection kit (Beyotime, Haimen, Jiangsu, China) was used to measure the intracellular ROS levels in accordance with this method.

#### 4.7.8. Jc-1 Assays, TUNEL Assay and Immunohistochemistry

The JC-1 Mitochondrial Membrane Potential Kit (Beyotime, Shanghai, China) and TUNEL BrightRed Apoptosis Detection Kit (Vazyme, Piscataway, NJ, USA) were used to detected the quantity of JC-1 and apoptotic cells, respectively. Cell that were treated with PPI for 24 h were subjected to both the Jc-1 assay and TUNEL assay. Primary antibodies against anti-γH2AX and anti-Rad51 were used for immunohistochemistry based on published methods [12].

#### 4.7.9. Western Blot Analysis

Cells were treated with 400 µg/mL PPI for 12 h in order to produce cell pellets for western blot analysis [41]. The following primary antibodies were used: anti-Rad51 (Abcam ab88572, London, UK), anti-γH2AX (Abcam ab26350, London, UK), anti-Bcl2 (ImmunoWay YT0470, Suzhou Jiangsu, China), anti-BAX (Cell Signaling Technology, #2772, Boston, MA, USA) and β-action (Abcam ab8227, London, UK).

#### 4.7.10. Bioinformatics Analysis of the mRNA Expression Profile

RNA extraction and RNA expression profiling were performed based on pre-existing methods [42]. GenePix 4.1 software (Molecular Devices, Sunnyvale, CA, USA) was used to analyse to microarray signal intensity of each spot. Three replicates in one group were used.

The expressions of mRNAs with log2 |fold change| > 1 (absolute |fold change| > 2) and *p* < 0.05 were defined as differentially expressed mRNAs. Hierarchical clustering analysis combined with a heatmap was applied to evaluate the three samples within each group and the differences between the two groups. An average linkage hierarchical clustering was generated using the R program in the heatmap.2 package, a function of g-plots package that is used for hierarchical clustering. Gene ontology analysis was performed using DAVID, a database for gene functional annotation.

#### 4.7.11. Statistical Method

The differences between mean values were statistically tested using Student’s t test or one-way ANOVA followed by the Tukey test for multiple comparisons. Comparisons were considered significant at *p* < 0.05 and *p* < 0.01 (asterisk).

## Figures and Tables

**Table 1:** Analysis of variance of the experimental results of the BBD (*** *p* < 0.001).

Source	Sum of Squares	Degree of Freedom	Mean Square	F Value	*p* Value
Model	60.38	9	6.710	89.09	<0.0001 ***
X_1_	4.96	1	4.961	65.88	<0.0001 ***
X_2_	13.65	1	13.650	181.26	<0.0001 ***
X_3_	19.75	1	19.750	262.25	<0.0001 ***
X_1_ × X_2_	6.55	1	6.550	87.02	<0.0001 ***
X_1_ × X_3_	0.06	1	0.058	0.76	0.4108
X_2_ × X_3_	6.79	1	6.790	90.11	<0.0001 ***
X_1_^2^	4.98	1	4.980	66.15	<0.0001 ***
X_2_^2^	3.19	1	3.190	42.34	<0.0003 ***
X_3_^2^	0.02	1	0.015	0.20	0.6685
Residual	0.53	7	0.075		
Lack of Fit	0.27	3	0.091	1.43	0.3586
Pure Error	0.25	4	0.064		
Cor Total	60.91	16			
R^2^ = 0.99	R^2^_Adj_ = 0.98	R^2^_pred_ = 0.92	Adeq Precisior = 33.86		

**Table 2:** Box-Behnken experimental design and the results for the Iron content of polysaccharide iron complex.

Run	Level			
	X_1_	X_2_	X_3_	Fe (%)
1	0	0	0	26.27
2	1	1	0	27.90
3	0	1	1	23.88
4	0	1	−1	29.86
5	1	0	−1	27.55
6	1	0	1	24.40
7	0	0	0	26.80
8	−1	0	1	23.35
9	0	0	0	26.20
10	0	−1	1	23.84
11	1	−1	0	22.76
12	0	−1	−1	24.61
13	0	0	0	26.2
14	−1	1	0	23.48
15	−1	−1	0	23.46
16	0	0	0	26.32
17	−1	0	−1	26.02

**Table 3:** Levels and code of extraction variables used in Box–Behnken design.

Variable	Symbols		Coded Levels	
	Coded	−1	0	1
pH	X_1_	7	8	9
Reaction temperature	X_2_	50	60	70
Sodium Citrate tribasic/Polysaccharide ratio	X_3_	0.5	1.25	2

## Data Availability

The data presented in this study are available in the article.

## Sample Availability

Samples of the compounds are not available from the authors.
